# Taking a complexity perspective when developing public health guidelines

**DOI:** 10.2471/BLT.19.230987

**Published:** 2019-04-01

**Authors:** Anayda Portela, Özge Tunçalp, Susan L Norris

**Affiliations:** a Department of Maternal, Newborn, Child and Adolescent Health, World Health Organization, avenue Appia 20, 1211 Geneva 27, Switzerland.; b Department of Reproductive Health and Research, World Health Organization, Geneva, Switzerland.; c Department of Information, Evidence and Research, World Health Organization, Geneva, Switzerland.

The Seventy-first World Health Assembly in 2018 approved the World Health Organization’s (WHO’s) thirteenth general programme of work, which is mostly based on the sustainable development goals (SDGs).^1^ Through this programme of work, WHO commits to supporting countries in reaching the SDGs, particularly the health-related SDG 3. Such support will require strategies that address primary health care, universal health coverage, underlying determinants of ill health and inequity, as well as complex challenges such as climate change, conflict and health emergencies.

To meet these goals, policy-makers and programme managers strive to make evidence-informed decisions for strategies and interventions, while dealing with competing sectoral priorities and constrained resources. Policy-makers need to consider the efficacy and safety of clinical interventions as well as information addressing health-system and health promotion strategies. Programme managers are interested in understanding how interventions interact with and can bring about wider changes in the health system as a whole.^2^ Often, knowing which interventions to implement is not enough, as countries also request guidance on how to implement either singular interventions or bundles of interventions.

Current evidence synthesis and guideline development methods can be enhanced to respond to these needs. Developers of evidence-informed guidance, including WHO, often apply processes and methods grounded in linear models of cause and effect^3^ that were adopted from the evidence-based medicine movement.^4^ However, these processes do not adequately consider relevant aspects of complexity in health interventions, including the multiple-component nature of some interventions, the nonlinear causal pathways to effects, and the relationship with the local context and how interventions interact within that context. The complex systems where these interventions are delivered and the diverse and often difficult-to-measure outcomes at the individual, population and/or system levels, are often not sufficiently examined either.^2^

Several international groups are actively working to advance the methods for assessing and synthesizing evidence and formulating recommendations on complex interventions.^5^^–^^8^ For example, a working group of the Grading of Recommendations Assessment, Development and Evaluation (GRADE) approach is developing methods for assessing the certainty of the body of evidence for health and social interventions, considering different dimensions of complexity.^9^

WHO has a unique position as a science- and evidence-based organization that sets global norms and standards.^1^ Therefore, WHO’s methods and processes for establishing global evidence-based guidance needs to reflect the latest methodological advances.^10^ WHO has initiated a collaborative effort to further advance the application of a complexity perspective to systematic reviews and to all stages of guideline development in the fields of public health and health systems. This effort is captured in a recently published supplement.^11^ The papers in this supplement address the major steps in the WHO guideline development process including: scoping and conceptualization of the guideline, formulating the priority research questions, synthesizing the quantitative, qualitative and mixed-methods evidence, and rating the quality of the body of evidence and the evidence-to-decision framework, where all criteria relevant to a health decision are systematically considered.

The supplement introduces the new WHO-INTEGRATE evidence-to-decision framework,^12^ which incorporates WHO’s norms and values with a complexity perspective. The framework aims to expand the deliberations on the key criteria to consider in health decision-making, such as balance of health benefits and harms, human rights and sociocultural acceptability, health equity, equality and non-discrimination, societal implications, financial and economic considerations and feasibility and health system considerations. This work is intended to strengthen WHO’s processes and methods, leading to more comprehensive evidence syntheses and impactful guidelines that respond to relevant issues faced by policy-makers and that highlight information applicable to varied contexts, therefore facilitating adaptation and implementation at the country level. Furthermore, we hope to stimulate rigorous primary research on a broad range of policy-relevant questions.

Work is ongoing to translate the findings of this series into pragmatic guidance for WHO guideline developers. We look forward to further collaboration with academics, researchers, programme implementers and other end-users to continue to apply and improve these methods. This will ensure that WHO guidelines remain updated and relevant, and most importantly, provide the much-needed normative guidance to support countries to achieve their goals for social transformation and improved health of their populations.
